# Drivers and Ideal Types towards Energy Transition: Anticipating the Futures Scenarios of OECD Countries

**DOI:** 10.3390/ijerph16081441

**Published:** 2019-04-23

**Authors:** Taewook Huh, Kee-Young Yoon, I Re Chung

**Affiliations:** 1 Moon Soul Graduate School of Future Strategy, Korea Advanced Institute of Science & Technology (KAIST), Daejeon 34141, Korea; 2 Department of Public Administration & Graduate School of Governance, Sungkyunkwan University, Seoul 03063, Korea; synsaje@gmail.com; 3 Graduate School of Environmental Studies, Seoul National University, Seoul 08826, Korea; chung2re@gmail.com

**Keywords:** energy transition, futures scenarios, OECD countries, fuzzy-set ideal type analysis

## Abstract

This study aims to identify the ideal types of energy transition of the thirty-five Organization for Economic Cooperation and Development (OECD) countries and to explore their implications using the fuzzy-set ideal type analysis. It then anticipates the futures scenarios of OECD member countries towards energy transition by placing the ideal type results. In particular, looking at the possibility of the futures towards energy transition, this study attempts to set up the comprehensive measurement framework of energy transition embracing the three key drivers (energy system (E), energy citizenship (S), and digital technology (T)). As a result, the eight OECD countries, including Denmark (fuzzy score 0.889), UK (0.800), and Norway (0.788) belonging to Type 1 (E*S*T) with the all high features of three key drivers, are expected to have ‘Outlier (super-potent) Futures’ of energy transition. The twelve countries of Type 2 (E*S*t), 3 (E*s*T), and 5 (e*S*T) with two high features of three ones will belong to the ‘Best (reformative) Futures’. The five countries of Type 4 (E*s*t), 6 (e*S*t), and 7 (e*s*T) with one high feature among three ones will be located in ‘Business-As-Usual Futures’. Finally, the ten countries, including Hungary (fuzzy score 0.881), Greece (0.716), Israel (0.679) belonging to Type 8 (e*s*t) with all three low features, are expected to have ‘Worst (declined) Futures’ of energy transition.

## 1. Introduction

In recent years, various efforts have been made to expand policies aimed at transitioning to sustainable systems, since there is a perception that the current socio-technical system is unsustainable as it uses enormous amounts of energy and emits carbon dioxide in the atmosphere [1,2,3]. In particular, the Organization for Economic Cooperation and Development (OECD) countries have attempted to set the specific policy objectives for the transition of social and technological systems [4,5]. For instance, the Dutch government has introduced the system transformation strategy called ‘Energy Transition’ to a sustainable society in order to address not only new energy technologies but also the changes in social systems where those technologies are produced and utilized [6,7].

The transition that the socio-technical system theory focuses on refers to a gradual and continuous structural transition process, which takes place in a given social system for more than one generation (around 30 years), and this transition is fundamentally based on multi-level perspectives and trans-disciplinary approach [8,9]. Lately, in terms of scrutinizing and theorizing the systemic properties of energy transition, a theoretical approach called ‘Transition Studies’ has been drawing attention as it analyzes and examines the changes and relevant policies of a socio-technical system [4,10,11]. As one of the major fields in the socio-technical system theory, the energy transition refers to a transition from supply-oriented and centralized energy system for fossil fuels and nuclear power energy sources to the energy system for demand management, such as the expansion of support for renewable energy, energy saving and improvement of efficiency, and reduction of energy consumption [12,13].

On the other hand, there has been a criticism that the energy transition has not yet occurred, and even it appears that changes in the economic and social system are still far-off to achieve the energy transition [14]. According to the international comparative analysis of the Levelized Cost of Electricity (LCOE) conducted by the International Energy Agency (IEA), countries like South Korea and Japan, where the use of renewable energy has not been accompanied by economies of scale due to geographical limitations, have forecasted that the economics of renewable energy will be low, unlike the LCOE forecasts of other major energy economies in the United States and Europe [14,15,16].

Considering these arguments (pros and cons), this study highlights that the energy transition requires to reflect the long-term transformative aspects of the socio-technical system theory in which the political, economic, and social elements have been simultaneously changing and rearranging, and the aspect of the implementation of multilateral governance that involves the participation of various entities [13,17,18]. Also, this study notes that the energy transition is a futures-oriented policy that requires more than a generation to be progressed [7,18]. Specifically, it emphasizes that the futures goals of energy transition require a ‘transition pathway’ for individual countries in terms of their political, social and economic environments. In other words, in order to achieve the energy transition, it first needs to explore the possibility of the futures through interactions with concrete reality.

Looking at the possibility of the futures towards the energy transition, this study attempts to newly set up the comprehensive measurement framework of the energy transition, embracing the arrangements of the five detailed variables (‘Energy Transition Index’, ‘Civic Engagement’, ‘Community’, ‘ICT Development Index’, and ‘E-Participation Index’) [19,20] of the three designed drivers (energy system (E), energy citizenship (S), and digital technology (T)); and to compare all thirty-five OECD member countries’ cases. This study takes the fuzzy-set ideal type analysis (STATA 12.0 (StataCorp LLC, College Station, TX, ISA) was used), which is characterized by a qualitative comparative research methodology to deal with middle-N cases that the existing comparative case analysis and regression analysis could not address. In short, this paper explores the following research questions.

- How do the ideal types of the energy transition in the OECD countries form, and what implications do they have in the context of energy transition?
- How do the futures scenarios of OECD countries towards the energy transition be anticipated by applying the results of fuzzy-set ideal type analysis.

This study has a different significance from other related studies in that it draws the futures scenarios of energy transition by utilizing the concrete evidence values (the fuzzy membership scores), which are the results of the fuzzy-set ideal analysis. In particular, it adopts the Schwartz’s future scenario theory with the four futures scenarios: ‘worst’ (declined), ‘Business-As-Usual’ (BAU), ‘best’ (reformative), and ‘outlier’ (super-potent) [21,22]. All thirty-five OECD countries were selected as the cases by extracting related data (variables) on the five variables newly composed by this study.

This paper is organized as follows: in Section 2, it discusses the theoretical background and issues of the energy transition, community energy, and energy citizenship in the socio-technical transition; of digital technology and energy blockchain; and of futures scenarios in the context of energy transition. In Section 3, it explains the application of the fussy-set analysis methodology and sets up the measurement frame works including the composition of categories and detailed variables, and the four futures scenarios based on the ideal types results of energy transition. In Section 4, it presents the findings of fussy-set ideal type analysis, and finally the conclusions on anticipating the futures scenarios of OECD case countries will be summarized in Section 5.

## 2. Theoretical Background and Issues

### 2.1. Energy Transition and Citizenship in Socio-Technical Transition

In recent years, there has been a growing interest in ‘socio-technical transition’, which deals with the phenomena of transition, system transition, system innovation, and sustainability transition. This theory is characterized by a long-term, structured approach to societal challenges, unemployment, inequality (polarization), and energy and environmental problems that continuously affect society and cannot be resolved in the short term [23]. This approach is very appropriate one in that a number of important social problems occurred in developed or/and developing countries are mainly due to the structural limitations of the socio-technical system [24,25].

In particular, the socio-technical transition theory advocates the transition to a socio-technical system composed of new technologies, activities, infrastructure, and markets to solve social challenges such as environment and energy. The transition theory focuses on producing policies and projects to achieve a transition with a long-term perspective of twenty to thirty [4,10]. For example, the energy transition is a major area of system transition policy. It pursues a socio-technical system that balances the production and consumption through the management of energy consumption, not just considering a one-sided policy of energy supply under the energy production-consumption structure. The energy transition requires the recognition and practices of energy consumers on the necessity of energy transition as well as a technological transition. For this reason, the implementation of multilateral governance involving various entities and actors should be premised throughout the ‘transition pathway’ (process) [24,25].

This study highlights that the energy transition requires a transition to new social systems, including energy production and consumption methods, energy subjects (actors), values, and governance, which are accompanied by changes in energy sources and technologies [13,18]. In other words, the energy transition requires a fundamental change to alter the path dependency and inertia of the socio-technical system that supports the centralized energy system, such as nuclear power plants and coal power plants [17,26]. It suggests that it is not only a matter of energy technology and physical infrastructure, but also a change in governance, including new market rules, laws and systems, decision-making systems, education and training, and transfer and coordination of resources among various actors. The energy transition is closely related to the decentralized energy system as the hierarchical market structures centered on a few capital-intensive, and this makes large corporations to be replaced by multiple small-scale generations and electric grids.

In this context, there is more attention paid to community energy potential as a ‘strategic niche’ (important practical means to expand the scope of a new system) for the energy transition [4,8,14]. The conception of community energy refers to creating energy policies based on energy conservation and efficiency improvement in the local areas, producing energy by directly utilizing renewable energy sources, and raising the energy self-reliance [27]. It can improve a democratic decision-making process and control over the energy for local people by allowing them to participate in discussions on local energy issues. In addition, since local residents can become employed as direct producers involving the production activities of energy, the costs and benefits of energy production can be circulated within the communities, which may in turn activate the local economies in the long run [28,29].

Meanwhile, the community energy is typically characterized by the community participation, ownership, and the sharing of profits in the energy transition pathway (process) [30]. In this sense, citizenship related to energy is a critical issue in discussions on the energy transition and the community energy as a strategic niche [31]. The energy citizenship can be recognized as a characteristic of citizens who are aware of the needs for active and socially reformative action and participation in the field of energy and climate change and also demonstrates their relevant [29,31]. The concept of energy citizenship intends to pursue a new future-oriented citizenship that is in line with deepening and expanding the existing conventional areas of democracy into the energy field, and thereby meeting the challenges of the energy transition [32]. In the future, eventually more citizens will emerge as the mainstay of energy production, storage, and supply. They can be called ‘energy citizens’ or ‘energy prosumers’ (consumer + producer) [33,34].

### 2.2. Digital Technology and Energy Blockchain in Energy Transition

Technological development in ‘the Fourth Industrial Revolution’ (https://www.weforum.org/about/the-fourth-industrial-revolution-by-klaus-schwab)—a new revolutionary era of ‘super-connection’ and ‘super-intelligence’ as a result of the conversion of the manufacturing industry and ICT, a term advocated by Klaus Schwab, the chairman of the World Economic Forum (WEF) in 2016—is expected to accelerate the transformation of energy systems and contribute to the creation of new services. Recently, ‘digital transition’ has been actively discussed in the energy field as a concept to explain the convergence of ICT and electric network that is the core of energy system integration [35]. For example, the IEA defines the energy digital transition as the convergence of ICT and energy, that is, the digital and physical boundaries are resolved, and a major change is expected in the energy sector [15].

In particular, this study notes that recently much attention has been given to the blockchain technology, which can operate as a revolutionary catalyst in the energy digital transition. Blockchain is a type of distributed ledger technology, a list which continuously grows in form of distributed database designed for discretionary control by the system operator to be impossible [36,37]. The most important features of the blockchain are anonymity, dispersibility (decentralization), transparency, and complementarity (system stability) [38]. Because blockchain has a distributive structure based on ‘distributed ledger technology’, which gets stored in the computers of multiple independent transaction parties (anonymous). It can reduce transaction fees through P2P (peer to peer) transactions without the Trusted Third Party (TTP) and does not require the centralized organizations nor publically certified third party (TTP) who guarantees credibility (transparency) [34,38]. Therefore, the costs for operation and maintenance, security, and financial transactions of the existing centralized system can be reduced.

This blockchain technology has attracted attention as a major technology of the fourth industrial revolution and has a great influence on the energy sector [39]. Through the energy blockchain, it is expected to build a transparent energy trading system, spread new and renewable energy trade, and discover future energy industry [40]. Energy blockchains are also called ‘eco-chains’ and have the advantage of securing stability, transparency, immediacy, and cost efficiency by using blockchain technology in the energy field [37,39]. In the near future, it will be possible for anyone to produce, sell, and consume energy (known as ‘energy prosumers’) through the distributed chain technology of blockchain and the smart contract technology; also possible to apply the blockchain in the field of the renewable energy cryptocurrency (‘green coin’), and of the power trading and liquidation [41] In fact, the energy blockchain allows for automatic power trading between power suppliers and consumers. Energy production and sales can be recorded on a blockchain-based distributed ledger, allowing anyone to see the transaction details, which would enable transparent transactions. For example, in the US and EU developed countries, there are several leading cases of smart contracts of energy blockchain using Etherium, cryptocurrency: “Brooklyn Microgrid Project” and “SolarCoin” in United States, “Slock.it & RWE” in Germany, and so on [36,41].

The blockchain technology is suitable for trading of renewable energy in the form of a micro-grid that refers to a small electric and integrated energy system consisting of distributed energy resources and multiple energy loads [36]. Since the existing hard energy generation methods such as nuclear power, thermal power, and hydro power produce electricity in one place and trade a large amount of electricity; therefore, Power Exchange-type transactions are efficient. However, for the direct trade between producers and consumers of renewable energy, the concept of micro-grid is more suitable. In order to realize and extend the micro-grid, the distributed chain technology of blockchain and the smart contract technology will be one of the most appropriate technologies.

Specifically, the sectors of energy blockchain, which are currently utilized domestically and overseas, can be broadly categorized as follows [36,41]. First type is the peer to peer (P2P) power trading. It reduces the cost of electricity trading among individuals and utilizes the blockchain technology for transparent power trading. Second type is the electric vehicle (EV) charging and sharing. It can be used for EV charging and payment, which can bring about reliability and cost savings. Third type is the utilization of the energy data. It builds a new business model by sharing energy data in a blockchain. Fourth type is the energy sharing (for the development of developing countries). It utilizes blockchains in order to share renewable energy facilities in developing countries in need of energy supply. Final type is the carbon asset transaction. It introduces the blockchain technology to facilitate carbon asset trading, such as the carbon emissions trading. Among the five types of energy blockchains, the P2P power trading is currently the most emerging case. It is predicted that if the blockchain is introduced into the energy sector in earnest, the value chain of the energy industry will change dramatically [39]. As a result, it is expected that the energy transition will be able to take a quick turn in the near future [34].

### 2.3. The Theories of Futures Scenarios and Energy Transition

Futures uncertainty towards energy transition may be the space of possibilities [42,43]. This study underlines that such a method of extrapolative forecasting that predicts the future by extrapolating the past and current trends may not be used to deal with the future challenges. As the EU Joint Research Centre (http://forlearn.jrc.ec.europa.eu/guide/1_why-foresight/characteristics.htm) argued, foresight is a social process to create the possible alternative futures as well as a dialogue or discussion to achieve a desirable future [44]. Therefore, one of the ways to imagine and discuss about various alternative futures is the futures scenarios. A scenario is a predictive technique that helps to clearly show possible situations not only in detail but also as a whole that could arise in the future in a story format [45]. The focus of this method is describing the future rather than to predict it in detail [42].

Futures scenarios can be developed by using multiple future variables [46], or by using the Critical Futures Driver. In other words, four futures scenarios of 2 × 2 can be derived from two future variables [47,48,49]. Examples of representative studies include the futures scenarios derived from futures archetypes of ‘growth’, ‘collapse’, ‘transformation’ and ‘discipline’ at a macro level [50], as well as derived from a ‘business-as-usual’, ‘the best’, ‘the worst’, and ‘the outlier’ cases for entities and organizations [21,22], and finally ‘preferred future’, ‘disowned future’, ‘integrated future’, and ‘the outlier’ for post-structuralists [47].

There is a variety of methods deployed to develop the futures scenarios, and there are pros and cons in these methods. Inayatullah [47]’s Critical Futures Driver to develop the futures scenarios has been characterized as intuitive, although it is difficult to draw the future transformation. Also, there are some limitations associated with the establishment of future strategies if future drivers are determined based on futures scenarios. Specifically, Dator [50]’s method of futures archetype can build a comprehensive strategy for the future, but it is difficult to lead the discussion about the futures of a certain culture, organization or country. In addition, the method of developing the futures scenarios in Post-structuralism allows a more contextual way of exploring the future images based on postmodernism, but it involves a range of stakeholders’ participatory approaches.

Therefore, this study attempts to employ the Schwartz [21,22]’s theory of scenario planning for entities, since it, unlike the other scenario studies above, has the strengths of clarity in ‘planned prior learning’ and of simplicity for the strategic decision making. The four scenarios (‘business-as-usual’, ‘the best’, ‘the worst’, and ‘the outlier’) allow for a clear approach, in setting the futures alternatives at the present moment as well as the conditions and variables towards the futures alternatives. In short, this study adopts the Schwarz ’s futures scenarios to describe the alternative futures in a more clear direction, which is considered to be the most appropriate method for developing the futures scenarios towards energy transition through a comparative fuzzy-set analysis of OECD member countries. Specifically, futures scenarios based on the three key drivers of the energy transition, such as energy system (E), energy citizenship (S), and digital technology (T) will be anticipated in this study. Based upon the Schwartz’s futures scenario theory, this study suggests the following four futures scenarios: ‘the worst’ (declined) scenarios where all three key drivers of energy transition are fragile; ‘business-as-usual’ (BAU) scenarios for the continuation of the current trend of energy transition; ‘the best’ (reformative) scenarios in which two of the key drivers of the energy transition arranged and appear positive; and ‘the outlier’ (super-potent) where all of the three drivers are jointly arranged and appear far more innovative than current situations (more detailed explanation in Section 3.2 below).

## 3. Methodology and Measurement Framework

### 3.1. Application of Fuzzy-Set Ideal Type Analysis Method

The fuzzy-set analysis (methodology) is a special form of the case study methodology known as qualitative comparative analysis (QCA) suggested by Zadeh from the University of California Berkeley in 1965 [51], and it has been applied in diverse ways by scholars such as Ragin and Kvist in social sciences [52,53]. The fuzzy-set analysis is the improved version of the methodology from qualitative comparative analysis [54,55] that has been previously used in social sciences. Going beyond the permission of the existing traditional two membership scores, 1 or 0, by using crisp set/set theory, the utilization of fuzzy-set, which has various membership scores between 0 and 1, can present not only the partial memberships but also the difference of the degree.

In particular, the fuzzy-set analysis has the following four major advantages. First, through exercising the fuzzy-set methodology, disadvantages of case-oriented and variable-oriented studies can be eliminated. The fuzzy-set analysis categorizes cases by a combined method of two strategies that variable-oriented quantitative methodology and qualitative case study, and it distinguishes itself from the existing analysis/methodology by examining a social diversity through comparative study [52,54]. Second, the fuzzy-set analysis allows to include the middle-case studies (15–25 cases), since both comparative case analysis and regression analysis could not address as being substantial subjects to analysis; however, it makes middle-class comparative analysis possible [52,56]. Moreover, it is also used to analyze joint causal relations by considering the interactive effects between each quality in a given case [53]. Third, it can explain diverse social phenomena. The fuzzy-set analysis overcomes the dichotomy method of 0 and 1 that have been used in many social science studies by enabling representation of various degrees between the 0 and 1, which minimizes the loss of information in analysis [57]. Fourth, it enables a more theoretical approach to categorization of types. The fuzzy-set analysis determines the number of memberships by categorizing the standards, and these standards consist of the ideal type formulated based on the theoretical background [58]. Accordingly, many recent studies are applying the fuzzy-set analysis to categorize individual ideal types [59,60].

This research categorizes all thirty-five OECD countries through comparative analysis by utilizing the fuzzy-set ideal type analysis. The fuzzy-set ideal type analysis is determined by fuzzy membership scores based on the fuzzy-set theory to demonstrate how close the subject of analysis is and is converted into fuzzy sets [61,62]. Through this process, it analyzes the degree of memberships for each category, converting the results of the existing original data into fuzzy-set membership scores. As the number of the sets is decided by the ideal type based on the fuzzy-set ideal type analysis unlike the existing cluster analysis, this research delivers a more systematic categorization and interpretation results [59,60].

The criteria for interpretation of membership scores of the fuzzy-set ideal type analysis drawn from this research is based on the theory suggested by Ragin (2008) [55]. In particular, as this research converted the scores into fuzzy-set score system through the calibrate function of STATA 12.0 (StataCorp LLC, College Station, TX, ISA), this study has measured them according to three qualitative anchors: ‘fully in’, ‘fully out’, and ‘crossover point’ as in the degree of the two. In other words, any score that is higher than the crossover point (0.5) is given strong membership (in the case the degree of full membership the given value possesses (FI: fully in or full membership) is higher than 95% (0.95)), and any score below the crossover point is given low membership score (in the case the degree of full membership is not present (FO: fully out or full non-membership) is lower than 5% (0.05)). The formula for calculating the Degree of Membership Score in the fuzzy-set ideal type analysis is as follows:

*Degree of Membership* = *exp*(*log odds*)/(1 + *exp*(*log odds*).

### 3.2. Measurement Frameworks

The socio-technical system transition theory that this study underlines (described in Section 2.1 above) is composed of multiple layers [24,25]. The energy transition corresponds to the long-term macro trend (landscape), and social, technical, and institutional conditions and norms that drive the energy transition belong to the meso-level. As described in Table 1 below, this study attempts to create the measurement framework to take a look at the meso-level of the energy transition by selecting the five detailed variables of the three variable categories (E: energy system, S: energy citizenship, T: digital technology, the constitutions of meso-level). The first E (Energy) category is characterized by the detailed variables of ‘Energy Transition Index’ (ETI), developed by World Economic Forum (WEF). The ETI emphasizes both the delivery of the energy system, including ‘secure and reliable access to energy’ and ‘environmental sustainability’, and the transition readiness (futures preparedness of countries’ systems), including ‘effective regulation and political commitment’, ‘stable institutions and governance’, and ‘supportive infrastructure’ [20]. The second S (Society) category consists of the both detailed variables ‘Civic Engagement’ (including ‘stakeholder engagement for developing regulations’ and ‘voter turnout’) and ‘Community’ (encompassing ‘quality of social support network’), which are from the Better Life Index of OECD (http://www.oecdbetterlifeindex.org). Both of the detailed variables are included in light of the context of the energy citizenship and the community energy, explained in Section 2.1.

The third T (Technology) category is composed of the both detailed variables ‘ICT Development Index’ (IDI) from the International Telecommunication Union (ITU) and ‘E-participation Index’ from United Nations. The former includes ‘ICT readiness’, the level of networked infrastructure and access to ICTs; ‘ICT use’, the level of intensity of ICTs in the society; and ‘ICT impact’, the results of ICT use [19]. The latter is with ‘e-information’, providing citizens with public information and access; ‘e-consultation’, engaging citizens in contributions to public policies and services; and ‘e-decision-making’, empowering citizens through co-design of policy option and co-production (https://publicadministration.un.org/egovkb/en-us/about/overview/e-participation). Two of the detailed variables are in line with the context of the digital transition and blockchain technology in energy transition, described in Section 2.2.

In order to conduct the fuzzy-set ideal type analysis, this study first weighted and standardized the five detailed variables and placed them in each of the three categories of energy transition. Second, it converted (calibrated) the three category variables into fuzzy scores by utilizing the three anchors (minimum, median (p50), and maximum) respectively, to identify the types of energy transition in all thirty-five OECD countries.

The degree of membership in this research was calculated and interpreted by the ‘principle of negation’, the ‘minimum principle’, and the ‘maximum principle’. This research sets the three category variables: ‘Energy System’ (Energy: E), ‘Energy Citizenship’ (Society: S), and ‘Digital Technology’ including energy blockchain (Technology: T). In this case, the principle of negation enables setting up negative categories of ‘e’, ‘s’, and ‘t’ through ‘1-Fuzzy-set membership score of the applicable category’. Accordingly, the ideal type was determined by applying the number of cases that each category variable can take, and this research postulated the eight ideal type sets (high or low) based on the three category variables (see Table 2).

In particular, these eight ideal type sets were yielded and interpreted by the ‘minimum principle’ and the ‘maximum principle’ [60,61]. The ‘minimum principle’ states that it is the minimum value among the fuzzy-set scores drawn from the principle of the eight types of ideal type categorization that will be the fuzzy-set membership score of the respective categories. In other words, among the fuzzy scores of the three variables (E, S, T) that consist the corresponding category sets, the minimum value will be selected. For example, if the fuzzy score of E in Category ‘E*S*T’ appears to be the minimum value, the fuzzy-set membership score of Category ‘E*S*T’ will be denoted as the fuzzy score of ‘E’ itself. Moreover, the ‘maximum principle’ postulates that while the fuzzy-set membership score of thirty-five OECD countries can conclusively be presented by eight types of categories, one with the maximum value of the membership score will be the category for the corresponding area.

**Figure 1:**
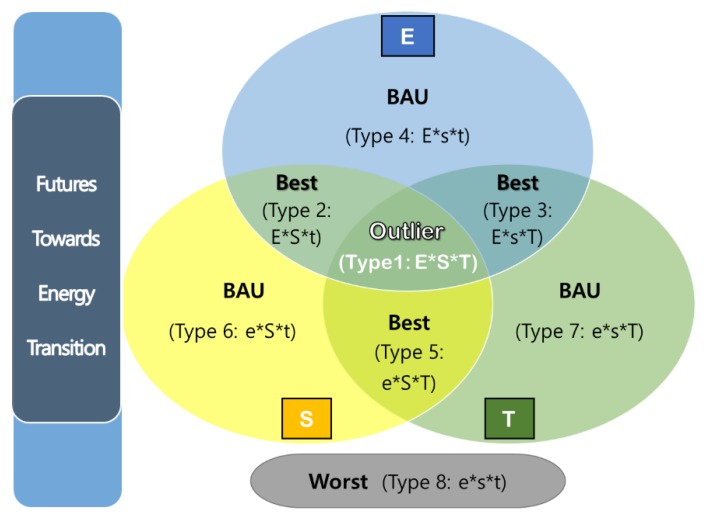
Diagram of four futures scenarios based on the eight ideal types of energy transition.

As a result, this study attempts to anticipate futures scenarios of OECD countries towards energy transition by adopting the Schwartz’s futures scenarios, as illustrated in Figure 1 above. It places each fuzzy-set ideal type in the four futures scenarios in which the relevant features of three key drivers (E: energy system, S: energy citizenship, and T: digital technology) are reflected. In short, Type 1 (E*S*T) with all three high features of key drivers is located in the middle overlapping of three ellipses, which means ‘Outlier (super-potent) Futures’ of energy transition. Type 2 (E*S*t), 3 (E*s*T), and 5 (e*S*T) with two high features of three key drivers respectively belong to the overlapping are of two ellipses, ‘Best (reformative) Futures’ of energy transition. Type 4 (E*s*t), 6 (e*S*t), and 7 (e*s*T) with one high feature among three ones respectively are included in ‘Business-As-Usual (BAU) Futures’ of energy transition, and finally Type 8 (e*s*t) with all low features is located in ‘Worst Futures’ of energy transition.

## 4. Findings and Results

Through the fuzzy-set ideal type analysis, the eight ideal types with the arrangement of the three variables (‘Energy System’ (E), ‘Energy Citizenship’ (S), and ‘Digital Technology’ (T)) are derived in of all thirty-five OECD member countries. Table 3 below shows the results of the fuzzy membership scores (fuzzy score) of the OECD countries for the sets of three variables (E, S, T) in this study—the fuzzy scores for each of the types in bold and shaded.

This study specifically identifies that the eight OECD countries belong to Type 1 (E*S*T, ‘strong energy transition countries’) with all high features of Energy System, Energy Citizenship and Digital Technology: Denmark (fuzzy score 0.889), UK (0.800), Norway (0.788), Finland (0.788), Sweden (0.760), New Zealand (0.716), Germany (0.557), and Luxembourg (0.557). On the other hand, the ten countries are included in Type 8 (e*s*t, ‘lite energy transition countries’) with the features of all low energy system (e), energy citizenship (s), and digital technology (t): Hungary (fuzzy score 0.881), Greece (0.716), Israel (0.679), Poland (0.661), Latvia (0.622), Mexico (0.622), Czech Rep. (0.594), Slovenia (0.571), Italy (0.524), and Turkey (0.524).

In Type 2 (E*S*t) with both high features of Energy System (E), Energy Citizenship (S) and the low feature of digital technology (t), the four countries including Iceland (0.741) and Belgium (0.557) are involved. Also, the four countries including Ireland (0.547) and France (0.512) belong to Type 3 (E*s*T) with both high features of Energy System (E) and Digital Technology (T), and the low feature of energy citizenship (s). In Type 4 (E*s*t) with the high feature of Energy System (E), and both low features of energy citizenship (s) and digital technology (t), the three countries including Austria (0.524) are categorized.

In Type 5 (e*S*T) with the low features of energy system (e), and both high features of Energy Citizenship (S) and Digital Technology (T), the four OECD countries including Australia (0.679) and Estonia (0.533) are involved. Then, the one country, Slovak Rep. (0.653) belongs to Type 6 (e*S*t) with both low features of energy system (e) and digital technology (t), and the high feature of Energy Citizenship (S). Also, only one country, Korea (0.852) belongs to Type 7 (e*s*T) with both low features of energy system (e) and energy citizenship (s), and the high feature of Digital Technology (T).

**Figure 2:**
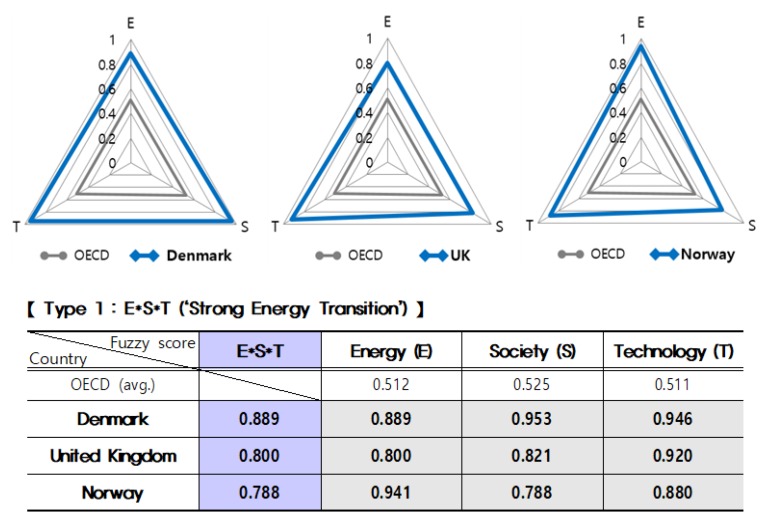
Comparison of main results by OECD countries: Type 1.

In addition, as illustrated in Figure 2 below, comparing the main results of fuzzy scores in both ‘strong’ and ‘lite’ ideal types (Type 1 and Type 8), Denmark (0.889), United Kingdom (0.800), Norway (0.788) belonging to Type 1 (E*S*T, ‘strong’) have very high scores in terms of the Energy System (E); also with high score results (0.953, 0.821, and 0.788 respectively) in the Energy Citizenship (S); and they record high scores as well (0.946, 0.920, and 0.880 respectively) in the Digital Technology (T).

These results of the three countries, the blue color triangles, are shown to be significantly large different from the OECD national average results, the grey color triangles.

**Figure 3:**
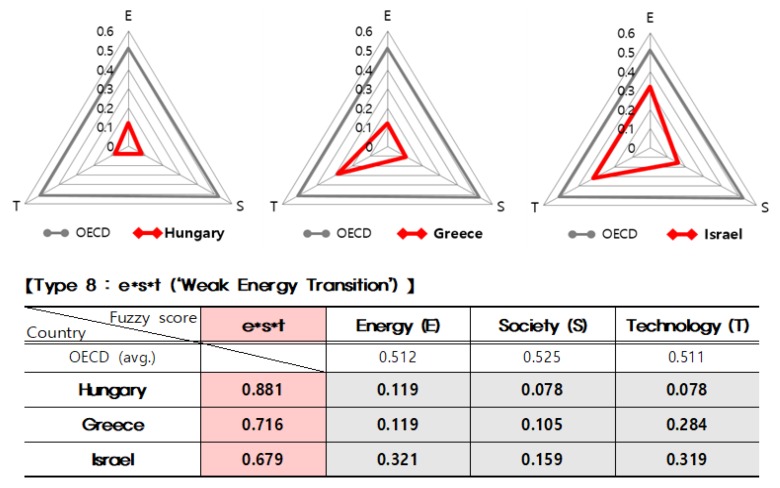
Comparison of the main results by OECD countries: Type 8.

On the other hand, as shown in Figure 3, Hungary, Greece, and Israel categorized in Type 8 (e*s*t, ‘lite’) remain very low (see Figure 3 below). In relation to the energy system (e), there were very low fuzzy scores such as 0.119 (Hungary), 0.119 (Greece), and 0.321 (Israel); also in the energy citizenship (s) with low score results (0.078, 0.105, and 0.159 respectively); and in the digital technology (t), they record low scores as well (0.078, 0.284, and 0.319 respectively). The results of three countries, the red color triangles, appear to be much narrower than the grey color triangles, which are the OECD national average results.

## 5. Conclusions and Implications: Deployment of Futures Scenarios

This study has attempted to set up the comprehensive measurement framework of energy transition embracing the arrangement of the three key categories (energy system (E), energy citizenship (S), and digital technology (T)) and the five detailed variables: ‘Energy Transition Index’, ‘Civic Engagement’, ‘Community’, ‘ICT Dev. Index’, and ‘E-Participation Index’. Then, it has compared all thirty-five OECD member countries’ cases through the fuzzy-set ideal type analysis. In short, the eight ideal types of energy transition for thirty-five OECD countries were deployed. Specifically, the eight OECD countries including Denmark (fuzzy membership score 0.889), UK (0.800), and Norway (0.788) belong to Type 1 (E*S*T), with the all three high features of energy transition. The ten countries including Hungary (fuzzy score 0.881), Greece (0.716), and Israel (0.679) are categorized in Type 8 (e*s*t) as having the all three low features.

**Figure 4:**
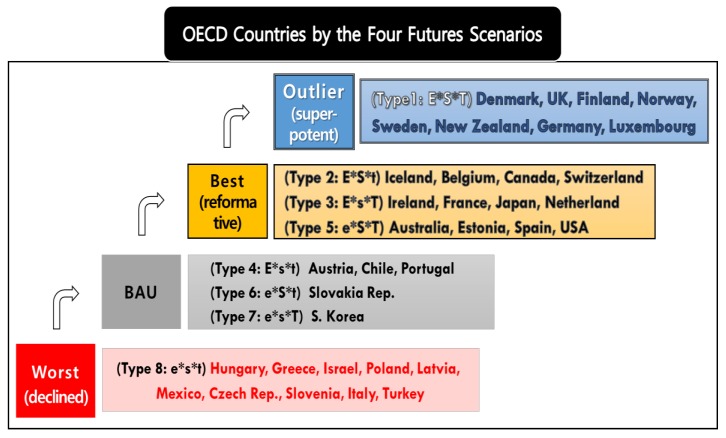
Futures scenarios results of energy transition in OECD countries.

Based on the measurement framework explained in Section 3.2, this study attempts to anticipates the four futures scenarios (‘worst’ (declined), ‘business-as-usual’ (BAU), ‘best’ (reformative), and ‘outlier’ (super-potent)) of OECD countries towards energy transition by placing the results of fuzzy-set ideal types. As a result, the eight OECD countries (Denmark, UK, Norway, Finland, Sweden, New Zealand, Germany, and Luxembourg) of Type 1 (E*S*T) with all high features of E (energy System), S (energy citizenship), and T (digital technology) are expected to have ‘Outlier (super-potent) Futures’ towards the energy transition, as shown in Figure 4 below.

The eight countries in this scenario will go through a transition pathway, in which they transform from fossil-fuel based societies and economies. Various ‘transition experiments’ will be broadened and scaled up in relation to new social systems, including energy production and consumption methods, energy citizenship, and governance, which are accompanied by a transformation in the energy digital transition. In addition, a decentralized energy system by multiple small-scale generators and smart grids (and microgrids) will be realized as ‘strategic niches’ of community energy. ‘Energy prosumers’ in terms of renewable energy cryptocurrency, smart contracts, P2P (peer to peer) power trading, EV (electric vehicle) charging and sharing, and energy data utilization will appear in a multitude of ways and become universal in the societies of the eight countries. Small-scale microgrids and global large-scale power trading will take place in an energy blockchain ecosystem. For example, microgrid systems such as the Brooklyn Microgrid Project in the United States, where local residents install solar panels and sell surplus power; and also the real-time electricity production and transaction data are stored in blocks through the smart meter, and automatic electricity trading is performed globally through the smart contracts. Beyond a ‘private blockchain’ ecosystem (closed blockchain, in which only people verified by the authentication methods made by the network for entry), everyone can participate and expand into a ‘public blockchain’ ecosystem that can form and approve transactions on the network. As a result, citizens in these scenarios lead a society that demonstrates energy citizenship and breaks out of the fossil fuel-based economy.

In addition, the twelve countries (including Iceland, Belgium, Ireland, France, Australia, and Estonia) of Type 2 (E*S*t), Type 3 (E*s*T), and Type 5 (e*S*T) with both high features among three category variables, will be positioned in the scenario of ‘Best (reformative) Futures’. The twelve countries in this scenario address energy issues with the application of digital technology in an integrated manner. Transition experiments of a reforming socio-economic system are progressing, forming a transition pathway, and deepening. A transition to renewable energy, including solar energy, is rapidly taking place, and there is an achievement of grid parity (i.e., when the unit cost of production for renewable energy becomes equal to that of electricity generated from fossil fuels [27,65,66]. Issues such as the stability, scalability, and operational cost efficiency of blockchains are resolved; the energy blockchain ecosystem stably develops; and as a result, legal tender and cryptocurrency with a 1:1 value are made. A private blockchain, closed blockchain deepens and broadens to become a public blockchain. Decentralized power trading on the microgrid becomes routine, and CO_2_ emissions trading also starts to use energy blockchains, which becomes commonplace. As the smart grids of regional units of the countries concerned are linked to the Global Energy Interconnection, energy prosumers become active players at the center of these societies.

On the other hand, the five OECD countries (Austria, Chile, Portugal, Slovakia Rep., and S. Korea) will be in ‘Business-As-Usual (BAU) Futures’. They belong to Type 4 (E*s*t), Type 6 (e*S*t), and Type 7 (e*s*T) respectively, consisting of only one high feature among three category variables. The Business-As-Usual (BAU) scenario refers to a slow and stubborn transition. In the five countries in this scenario, the transition to renewable energy, such as solar energy, steadily deepens. The number of countries achieving grid parity increases but at a very moderate pace. The energy blockchain is gradually employed in the microgrid. A smart agreement of the blockchain are made and deepened, but an energy blockchain ecosystem remains at the level of a closed private blockchain. Though various examples of community energy grow, energy citizenship is still unable to be implemented or become mainstream. The per capita energy consumption of the citizens of such countries does not decrease significantly, and there are no major changes in the energy supply-oriented system.

The last ten countries, including Hungry, Greece, Israel, and Poland, shown in Figure 4 above, are expected to have ‘Worst (declined) Futures’. This is the futures scenario marked by the failure of energy transition or regression. The ten countries in this scenario can technologically achieve grid in 20-30 years, but under a centralized one-way energy system, there is still not enough increase in the share of renewable energy. The transaction costs for the energy blockchain ecosystem will remain too high compared to the power trading gains in the microgrid. While there are attempts to make alternatives to this system, stakeholders that include existing power generators harshly oppose them, and ordinary citizens do not show much interest and effort. Thus, a static energy supply-oriented social system is maintained.

Meanwhile, this study’s futures scenarios towards energy transition are advantageous as a strategic decision-making tool for environmental planning and policy, which can explore various paths, easily deliver predictions for each path as a story. In particular, the results of energy transition scenarios with a ‘transition pathway’ for individual OECD countries in terms of their political, social and economic environments through this study can be further developed into a more meaningful academic contribution in the field of related environmental studies. However, the anticipation of futures scenarios in this study suggests a rough context, and does not provide a detailed description of transition paths and experiments for each OECD country. In order to overcome these limitations, it is necessary to conduct in-depth analysis of more complex current and futures factors that lead to energy transition through further studies.

## Figures and Tables

**Table 1:** The variable framework of the fuzzy-set ideal type analysis.

	Categories		Detailed Variables	Weight	References (year)
Energy Transition	Energy (E)	Energy System	Energy Transition Index (ETI)	100%	World Economic Forum (2018) [20]
	Society (S)	Energy Citizenship	Civic Engagement	50%	OECD (2017) [63] ‘Better Life Index’
			Community	50%	
	Technology (T)	Digital Technology (energy blockchain)	ICT Dev. Index (IDI)	50%	Intl. Tele. Union (ITU) (2017) [19]
			E-Participation Index (EPI)	50%	United Nations (2018) [64]

**Table 2:** The eight ideal type sets.

Ideal Type	Features of Types
1: E*S*T	High Energy, Society, Technology for Energy Transition
2: E*S*t	High Energy, Society, & low technology for Energy Transition
3: E*s*T	High Energy, Technology, & low society for Energy Transition
4: E*s*t	High Energy, & low society, technology for Energy Transition
5: e*S*T	low energy, & High Society, Technology for Energy Transition
6: e*S*t	low energy, technology & High Society for Energy Transition
7: e*s*T	low energy, society & High Technology for Energy Transition
8: e*s*t	low energy, society, technology for Energy Transition

**Table 3:** Results of Fuzzy-set Ideal Type Analysis by OECD Countries.

	Type	1	2	3	4	5	6	7	8	Ideal Type
Country		E*S*T	E*S*t	E*s*T	E*s*t	e*S*T	e*S*t	e*s*T	e*s*t	
**Denmark**		**0.889**	0.054	0.047	0.047	0.111	0.054	0.047	0.047	Type 1: E*S*T
**UK**		**0.800**	0.080	0.179	0.080	0.200	0.080	0.179	0.080	
**Finland**		**0.788**	0.210	0.212	0.210	0.111	0.111	0.111	0.111	
**Norway**		**0.788**	0.120	0.212	0.120	0.059	0.059	0.059	0.059	
**Sweden**		**0.760**	0.240	0.212	0.212	0.047	0.047	0.047	0.047	
**New Zealand**		**0.716**	0.136	0.104	0.104	0.284	0.136	0.104	0.104	
**Germany**		**0.557**	0.310	0.359	0.310	0.443	0.310	0.359	0.310	
**Luxembourg**		**0.557**	0.220	0.249	0.220	0.443	0.220	0.249	0.220	
**Iceland**		0.259	**0.741**	0.109	0.109	0.240	0.240	0.109	0.109	Type 2: E*S*t
**Belgium**		0.199	**0.557**	0.157	0.157	0.199	0.443	0.157	0.157	
**Canada**		0.442	**0.502**	0.186	0.186	0.442	0.498	0.186	0.186	
**Switzerland**		0.498	**0.502**	0.461	0.461	0.090	0.090	0.090	0.090	
**Ireland**		0.453	0.421	**0.547**	0.421	0.334	0.334	0.334	0.334	Type 3: E*s*T
**France**		0.488	0.213	**0.512**	0.213	0.200	0.200	0.200	0.200	
**Japan**		0.153	0.116	**0.502**	0.116	0.153	0.116	0.498	0.116	
**Netherlands**		0.499	0.097	**0.501**	0.097	0.200	0.097	0.200	0.097	
**Austria**		0.332	0.476	0.332	**0.524**	0.200	0.200	0.200	0.200	Type 4: E*s*t
**Chile**		0.047	0.047	0.132	**0.502**	0.047	0.047	0.132	0.498	
**Portugal**		0.092	0.092	0.305	**0.502**	0.092	0.092	0.305	0.498	
**Australia**		0.321	0.157	0.058	0.058	**0.679**	0.157	0.058	0.058	Type 5: e*S*T
**Estonia**		0.378	0.378	0.378	0.378	**0.533**	0.467	0.384	0.384	
**Spain**		0.498	0.302	0.359	0.302	**0.502**	0.302	0.359	0.302	
**USA**		0.499	0.173	0.269	0.173	**0.501**	0.173	0.269	0.173	
**Slovak Rep.**		0.170	0.223	0.170	0.223	0.170	**0.653**	0.170	0.347	Type 6: e*S*t
**Korea**		0.135	0.047	0.148	0.047	0.135	0.047	**0.852**	0.047	Type 7: e*s*T
**Hungary**		0.078	0.078	0.078	0.119	0.078	0.078	0.078	**0.881**	Type 8: e*s*t
**Greece**		0.105	0.105	0.119	0.119	0.105	0.105	0.284	**0.716**	
**Israel**		0.159	0.159	0.319	0.321	0.159	0.159	0.319	**0.679**	
**Poland**		0.047	0.047	0.047	0.047	0.258	0.339	0.258	**0.661**	
**Latvia**		0.083	0.225	0.083	0.378	0.083	0.225	0.083	**0.622**	
**Mexico**		0.118	0.225	0.118	0.378	0.118	0.225	0.118	**0.622**	
**Czech Rep.**		0.047	0.148	0.047	0.148	0.047	0.406	0.047	**0.594**	
**Slovenia**		0.217	0.321	0.217	0.321	0.217	0.429	0.217	**0.571**	
**Italy**		0.321	0.321	0.321	0.321	0.388	0.476	0.388	**0.524**	
**Turkey**		0.095	0.095	0.095	0.095	0.124	0.476	0.124	**0.524**	
